# Identification of Bioactive Phytochemicals in Leaf Protein Concentrate of Jerusalem Artichoke (*Helianthus tuberosus* L.)

**DOI:** 10.3390/plants9070889

**Published:** 2020-07-14

**Authors:** László Kaszás, Tarek Alshaal, Hassan El-Ramady, Zoltán Kovács, Judit Koroknai, Nevien Elhawat, Éva Nagy, Zoltán Cziáky, Miklós Fári, Éva Domokos-Szabolcsy

**Affiliations:** 1 Department of Agricultural Botany, Plant Physiology and Biotechnology (MEK), Debrecen University, Böszörményi Street 138, 4032 Debrecen, Hungary; kaszas.laszlo@agr.unideb.hu (L.K.); ramady2000@gmail.com (H.E.-R.); kovacs.zoltan@agr.unideb.hu (Z.K.); koroknaij@agr.unideb.hu (J.K.); nevienelhawat@gmail.com (N.E.); nagyeva0116@gmail.com (É.N.); fari@agr.unideb.hu (M.F.); szabolcsy@agr.unideb.hu (É.D.-S.); 2 Soil and Water Department, Faculty of Agriculture, Kafrelsheikh University, Kafr El-Sheikh 33516, Egypt; 3 Department of Biological and Environmental Sciences, Faculty of Home Economic, Al-Azhar University, Tanta 31732, Egypt; 4 Agricultural and Molecular Research and Service Institute, University of Nyíregyháza, 4407 Nyíregyháza, Hungary; cziaky.zoltan@nye.hu

**Keywords:** circular economy, green biorefinery, polyunsaturated fatty acids, phytochemicals, amino acids, food and feed, UHPLC-ESI-ORBITRAP-MS/MS

## Abstract

Jerusalem artichoke (JA) is widely known to have inulin-rich tubers. However, its fresh aerial biomass produces significant levels of leaf protein and economic bioactive phytochemicals. We have characterized leaf protein concentrate (JAPC) isolated from green biomass of three Jerusalem artichoke clones, Alba, Fuseau, and Kalevala, and its nutritional value for the human diet or animal feeding. The JAPC yield varied from 28.6 to 31.2 g DM kg^−1^ green biomass with an average total protein content of 33.3% on a dry mass basis. The qualitative analysis of the phytochemical composition of JAPC was performed by ultra-high performance liquid chromatography-electrospray ionization-Orbitrap/mass spectrometry analysis (UHPLC-ESI-ORBITRAP-MS/MS). Fifty-three phytochemicals were successfully identified in JAPC. In addition to the phenolic acids (especially mono- and di-hydroxycinnamic acid esters of quinic acids) several medically important hydroxylated methoxyflavones, i.e., dimethoxy-tetrahydroxyflavone, dihydroxy-methoxyflavone, hymenoxin, and nevadensin, were detected in the JAPC for the first time. Liquiritigenin, an estrogenic-like flavanone, was measured in the JAPC as well as butein and kukulkanin B, as chalcones. The results also showed high contents of the essential amino acids and polyunsaturated fatty acids (PUFAs; 66-68%) in JAPC. Linolenic acid represented 39–43% of the total lipid content; moreover, the ratio between ω-6 and ω-3 fatty acids in the JAPC was ~0.6:1. Comparing the JA clones, no major differences in phytochemicals, fatty acid, or amino acid compositions were observed. This paper confirms the economic and nutritional value of JAPC as it is not only an alternative plant protein source but also as a good source of biological valuable phytochemicals.

## 1. Introduction

The global protein demand continuously grows as the world population exponentially increases. In Europe, the increasing protein dependency particularly obtained from soybean has triggered an urgent need for alternative production systems. Locally grown green biomass crops represent an alternative protein source. Due to high green biomass yield and regrown capacity, clover, alfalfa, and grasses are the most common and prospective plant species for leaf protein isolate. However, digestion of green biomass by monogastric animals is difficult because of its high fiber content [1]. Green biorefinery is a complex processing system with a dedicated goal of making a commercially viable production system of added-value protein based on green biomass [2]. Separating fresh green biomass into two fractions is a key step in the green biorefinery. The fibrous pulp contains insoluble and fiber-bound protein, while the other fraction (green juice) is soluble protein-rich [1]. Soluble proteins in green juice can be precipitated by different techniques. Recovered protein concentrate is separated from brown juice fraction by filtration. Moreover, the quality of leaf protein concentrate as a main product is very important. Based on extended qualitative and quantitative analysis, alfalfa leaf protein concentrate can be directed towards feed and/or food [3,4]. However, besides the well-known herbaceous species, a range of agro-industrial crops is constantly expanding, which can be utilized in the green biorefinery [5].

Jerusalem artichoke (JA), a perennial plant, belongs to the Asteraceae family. Cultivation of JA has many advantages as it is tolerant to biotic stress, i.e., pests and diseases [6]. It can grow normally in a wide range of soils including salt-affected soil, sandy soil, and marginal lands with nearly zero levels of fertilization [7,8,9]. Moreover, it showed potential resistance to drought, frost, and high temperatures [10]. It yields a huge green biomass almost 120 tons ha^−1^ fresh mass [11]. These aspects are important when avoiding competition with food production on arable lands. The recognized nutritional value of JA is mainly due to the high inulin and fructose contents in its tubers, which additionally contain protein, nutrients, and vitamins [12]. Additionally, JA is well-known as multipurpose use crop where its aerial part has attracted the interest of many researchers, firstly, concerning bioenergy production due to its high lignocellulosic content, high biomass yield, and low inputs [6]. Among the phytochemicals, sesquiterpene lactones, phenolic acids, flavone glucosides (kaempferol 3-*O*-glucoside and quercetin 7-*O*-glucoside), chlorophylls, and carotenoids have been described by several authors in the whole plant or different organs such as tubers, leaves, or flowers [13,14,15,16,17,18,19]. These isolated phytochemicals are known as potential anticancer, antidiabetic, antioxidant, antifungal, and antimicrobial in addition to their other medical uses [13,17]. 

Despite green leafy shoot of JA can be utilized directly as fresh forage, silage, or food pellets for animal feeding [9,12], most of the animal species do not prefer it because of trichome-rich leaves and stems [8]. Considering its high green biomass, regeneration capacity, and chemical composition, leafy shoots of JA can be alternatively used in the green biorefinery practice; however, there is a shortage of knowledge in this area [20]. 

The objectives of the present work were to produce and characterize the biological value of JAPC originating from the green biomass of JA. We aimed to provide detailed insights into the extraction efficiency and biochemical composition of JAPC. Therefore, three clones of JA representing different climatic zones were grown under low input conditions in Hungary. In addition to total protein, amino acid composition, and fatty acids profile the biochemical composition and qualitative determination of phytochemicals in the JAPCs from these clones were measured using ultra-high performance liquid chromatography-electrospray ionization-Orbitrap/mass spectrometry analysis (UHPLC-ESI-ORBITRAP-MS/MS).

## 2. Materials and Methods 

### 2.1. Experimental Installation

A field experiment was conducted in 2016 at the Horticultural Demonstration garden of the University of Debrecen, Hungary (47°33′ N; 21°36′ E). Three different clones of JA (i.e., Alba, Fuseau, and Kalevala) were compared for their fresh aerial biomass, phytochemical content, and biochemical traits of the JAPC, under low input conditions. Tubers of JA clones representing three climatic zones were obtained from different sources as follows: Alba was obtained from a Hungarian market; Fuseau was obtained from Ismailia, Egypt; and Kalevala was obtained from Helsinki, Finland. The experiment was set up in a randomized complete block design with six replicates. The area of the experimental plot was 0.8 × 0.6 m2; the row was 3.5 m in length and 0.8 m in width, and within-row spacing was 0.6 m. The cultivation of the JA clones started on 5 April 2016, using identically sized tubers (60–80 g/tuber). Neither irrigation nor fertilization was applied. The chemical characteristics of the experimental soil were as follows: total N (555 ± 2 mg kg^−1^); total P (6793 ± 17 mg kg^−1^); total K (1298 ± 7 mg kg^−1^); and humus (1.9% ± 0.02%).

### 2.2. Harvest of Above-Ground Biomass

Due to the ability of JA plants to regrow, the green biomass of the three clones was harvested twice during the growing season, when young shoots reached 1.3–1.5 m in height from the soil surface. The first harvest was conducted on 27 June 2016, and the second on 8 August 2016. The fresh yield of the aerial parts was measured.

### 2.3. Fractionation of Harvested Green Biomass

The harvest of JA plants was conducted early in the morning and they were immediately transferred to the laboratory in an icebox to prevent the chemical compounds from degrading. The plants were harvested 15–20 cm above the soil surface. A 1 kg harvest of green biomass was mechanically pressed and pulped using a twin-screw juicer (Green Star GS 3000, Toronto, ON, Canada) in three replicates. Thereafter, the green juice was thermally coagulated at 80 °C in one step to obtain the JAPC. The JAPC was separated from the brown-colored liquid fraction using cloth filtration. Both the fresh and dry masses of the JAPC were measured before it was lyophilized using an Alpha 1–4 LSC Christ lyophilizer.

### 2.4. Biochemical Composition of JAPC

#### 2.4.1. Crude Protein Content

The total protein content of the JAPC was measured as total N content using the Kjeldahl method [21]. Briefly, 1 g lyophilized sample was weighed in a 250 mL Kjeldahl digestion tube, then 15 mL concentrated sulfuric acid (99%, VWR Ltd., Debrecen, Hungary) and two catalyst tablets were added. The Kjeldahl digestion tubes were placed in a Tecator Digestor (VELT, VWR Ltd, Debrecen, Hungary) at 420 °C for 1.5 h. The total N content in the digested samples was measured by titration and calculated based on the weight of the titrated solution and the sample weight. The total protein content of the sample was calculated using the following equation: Total protein % = total N content × 6.25.

#### 2.4.2. Quantification of Amino Acid Composition in JAPC Using an Amino Acid Analyzer

Lyophilized and ground samples of JAPC were digested with 6 M HCl at 110 °C for 23 h. Since the digested sample was designed to contain at least 25 mg N, the measured weights of the samples were variable. Alternating application of inert gas and a vacuum using a three-way valve was conducted to remove air. Following hydrolysis, the sample was filtered into an evaporator flask and the filtrate was evaporated under 60 °C to achieve a syrup-like consistency. Thereafter, distilled water was added to the sample and evaporation was conducted twice more under the same conditions. The evaporated sample was washed with citrate buffer pH 2.2. For the analysis of amino acid composition an INGOS AAA500 (Ingos Ltd., Prague, Czech Republic) amino acid analyzer was used. The separation was based on ionic exchange chromatography with post-column derivatization of ninhydrin. A UV/VIS detector was used at 440/570 nm.

#### 2.4.3. Determination of Fatty Acid Composition in JAPC Using Gas Chromatography

The esterification of fatty acids in the JAPC fraction into methyl esters was conducted using a sodium methylate catalyst. Lyophilized homogeneous sample (70 mg) was weighed into a 20 mL tube; 3 mL of n-hexane, 2 mL of dimethyl carbonate and 1 mL of sodium methylate in methanol were added. The contents of the test tube were shaken for 5 min (Janke and Kunkel WX2) and then 2 mL of distilled water was added before the tube was shaken again. The samples were centrifuged at 3000 rpm for 2 min (Heraeus Sepatech, UK). A 2.0 mL sample of supernatant (hexane phase) was transferred into a container through filter paper, which contained anhydrous sodium sulfate. The prepared solution contained approximately 50–70 mg cm−3 fatty acid methyl ester (FAME) and was suitable for analysis by gas chromatography. Gas chromatography was performed using an Agilent 6890 N coupled to an Agilent flame ionization detector. A Supelco Omegawax capillary column (30 m, 0.32 mm i.d., 0.25 μm film thickness) was used to separate FAMEs. The oven temperature was 180 °C and the total analysis time was 36 min. An Agilent 7683 automatic split/splitless injector was used with an injector temperature of 280°C and a 100:1 split ratio. The injection volume was 1 μL. The carrier gas was hydrogen with a flow rate of 0.6 mL min−1 and the makeup gas was N with a flow rate of 25.0 mL min^−1^. The components were identified from retention data and standard addition.

### 2.5. Screening of Phytochemicals in JAPC by UHPLC-ESI-ORBITRAP-MS/MS

#### 2.5.1. Sample Preparation

To prepare the hydro-alcoholic extracts, 0.5 g ground JAPC powder was extracted with 25 mL methanol:water solution. The mixture was stirred at 150 rpm for 2 h at room temperature. The hydro-alcoholic extracts were filtered using a 0.22 µm PTFE syringe filter.

#### 2.5.2. UHPLC-ESI-ORBITRAP-MS/MS Analysis

Phytochemical analyses were performed using UHPLC-ESI-ORBITRAP-MS/MSwith a Dionex Ultimate 3000RS UHPLC system (Thermo Fisher, Waltham, MA, USA) coupled to a Thermo Q Exactive Orbitrap hybrid mass spectrometer equipped with a Thermo Accucore C18 analytical column (2.1 mm × 100 mm, 2.6 µm particle size). The flow rate was maintained at 0.2 mL/min and the column oven temperature was set to 25 °C ± 1 °C. The mobile phase consisted of methanol (A) and water (B) (both acidified with 0.1% formic acid). The gradient program was as follows: 0–3 min, 95% B; 3–43 min, 0% B; 43–61 min, 0% B; 61–62 min, 95% B; and 62–70 min, 95% B. The injection volume was 2 μL.

#### 2.5.3. Mass Spectrometry Conditions

A Thermo Q Exactive Orbitrap hybrid mass spectrometer (Thermo Fisher, Waltham, MA, USA) was equipped with an ESI source. The samples were measured in both positive and negative ionization modes separately. The capillary temperature was 320 °C and spray voltages were 4.0 kV in positive ionization mode and 3.8 kV in negative ionization mode, respectively. The resolution was 35,000 for MS1 scans and 17,500 for MS2 scans. The scanned mass interval was 100–1500 *m*/*z*. For the tandem MS (MS/MS) scans, the collision energy was set to 30 nominal collision energy units. The difference between measured and calculated molecular ion masses was less than 5 ppm in each case. The data were acquired and processed using Thermo Trace Finder 2.1 software based on own and internet databases (Metlin, Mass Bank of North America, *m*/*z* Cloud). After processing, the results were manually checked using Thermo Xcalibur 4.0 software (ThermoFisher, Waltham, MA, USA). 

### 2.6. Quality Assurance of Results

The glass- and plastic-ware used for analyses were usually new and were cleaned by soaking in 10% (v/v) HNO_3_ for a minimum of 24 h, followed by thorough rinsing with distilled water. All chemicals were analytical reagent grade or equivalent analytical purity. All equipment was calibrated, and uncertainties were calculated. Internal and external quality assurance systems were applied at the Central Laboratory of the University of Debrecen, according to MSZ EN ISO 5983-1: 2005 (for Total N), and the Bunge Private Limited Company Martfű Laboratory, according to MSZ 190 5508: 1992 (for fatty acid composition).

### 2.7. Statistical Analysis

Before the ANOVA test, Levene’s Test for Equality of Variances was performed. The Levene’s test for different variables at all treatments was negative, *p* < 0.05, showing homogeneity of the variances. The experimental design was established as a randomized complete block design with six replicates. The data obtained from the experiments were subjected to one-way ANOVA by ‘R-Studio’ software and the means were compared by Duncan’s Multiple Range Test at *p* < 0.05 [22].

## 3. Results

### 3.1. Green Biomass of Jerusalem Artichoke Clones

The yield of the aerial fresh biomass of different JA clones is presented in Table 1. Clones displayed almost the same fresh biomass yield. Hence, no significant differences among the clones (i.e., Alba, Fuseau, and Kalevala) were noticed, especially during the first harvest. The harvest time largely influenced the yield. The average fresh biomass yield was approximately 5.3 kg m^−2^ for the first harvest, while for the second harvest the yield was significantly reduced to 2.4 kg m^−2^ (Table 1). The total aerial fresh biomass yield—as an average—was estimated to be 7.7 kg m^−2^.

### 3.2. JAPC Yield

The yield of JAPC, extracted using thermal coagulation, from 1 kg fresh green biomass of the JA clones is displayed in Table 1. No significant differences were seen between the JA clones in either the first or the second harvests. The JAPC yield ranged from 28.3 (Fuseau) to 32.3 (Kalevala) g kg^−1^ fresh biomass for the first harvest, while for the second harvest it varied from 28 (Kalevala) to 30.4 (Alba) g kg^−1^ fresh biomass (Table 1). However, the results showed that the average JAPC dry yield from the first and second harvests was 30.8 and 29.1 g kg^−1^ fresh biomass, respectively. Therefore, 1 kg of green biomass of JA was estimated to yield approximately 30 g JAPC dry mass as an annual average.

### 3.3. Total Protein Content of JAPC

The total protein content (m/m%) of JAPC generated from fresh green biomass of JA clones ranged between 33.3 m/m% (Fuseau) and 35.3 m/m% (Alba) in the first harvest, while in the second, it varied from 31.6 m/m% (Alba) to 35.2 m/m% (Fuseau). Statistically, no significant differences were calculated either between the clones or harvests (Table 1). The average total protein content in the first harvest was 34.1 m/m% and 33.4 m/m% in the second based on the dry weight. The annual average total protein content of the JAPC extracted from the JA fresh biomass was estimated to be 33.8 m/m% (Table 1).

### 3.4. Amino Acid Composition of JAPC 

The amino acid composition of the JAPC obtained from the green biomass of the JA clones is presented in Table 2. Essential amino acids (i.e., lysine, histidine, isoleucine, leucine, phenylalanine, methionine, threonine, and valine) play a major nutritional role in feed; therefore, they are of special interest. Among the investigated JA clones, Kalevala displayed the highest content of five essential amino acids (i.e., phenylalanine, histidine, isoleucine, threonine, and valine). Additionally, the content of aspartic acid, glycine, glutamic acid, proline, and serine was the highest in Kalevala, with values of 4.23, 2.13, 4.82, 2.20, and 1.90 m/m%, respectively (Table 2). Lysine is particularly important in animal feed and its content in Alba, Fuseau, and Kalevala ranged between 2.19 and 2.32 m/m% in the first harvest. Lysine content in the clones was similar regardless of the harvest time with higher value in the second harvest (2.35–2.54 m/m%) than the first harvest. Methionine is another limiting essential amino acid. The methionine content in Alba and Fuseau clones ranged between 0.82 and 0.95 m/m% in both harvests (Table 2). A reduction in methionine content was found in the second harvest for all clones except Fuseau.

### 3.5. Qualitative Analysis of JAPC Fatty Acid Composition

**Figure 1:**
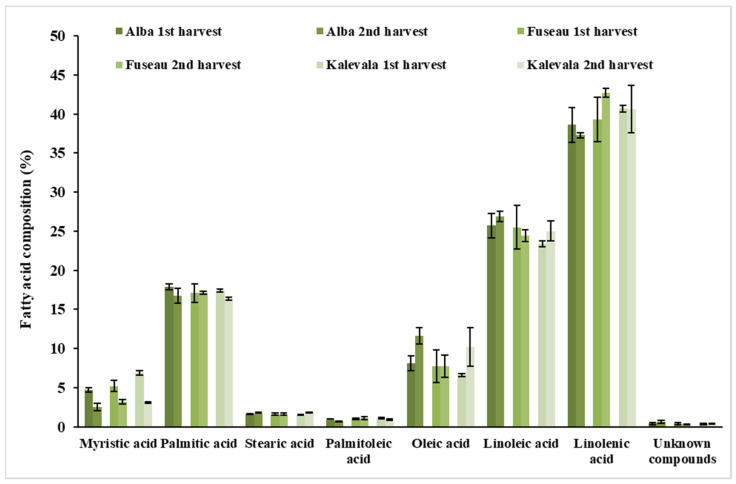
Fatty acid composition (%) of Jerusalem artichoke leaf protein concentrate (JAPC) extracted from the green biomass of three clones (Alba, Fuseau, and Kalevala).

**Figure 2:**
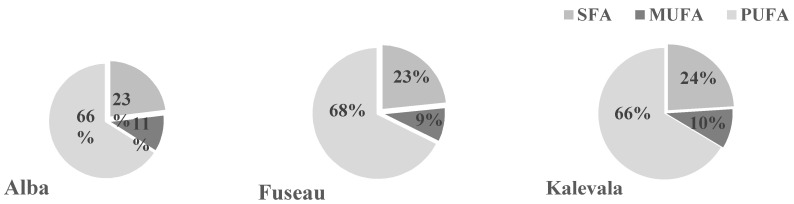
Distribution of saturated fatty acids (SFA), monounsaturated fatty acids (MUFA), and polyunsaturated fatty acids (PUFA) in Jerusalem artichoke leaf protein concentrate (JAPC) extracted from the green biomass of three clones (Alba, Fuseau, and Kalevala).

Both saturated (SFA) and unsaturated fatty acids (UFA) were detected in the JAPC. Polyunsaturated fatty acids (PUFA) including linoleic acid (C18:2ω –6) and linolenic acid (C18: 3ω –3) predominated (66%–68%) in all of the JA clones (Figure 1 and Figure 2). Among these fatty acids, linolenic acid (38.6%–42.7%) exhibited a narrow range of content that was present in the highest amount regardless of harvest time or clone. Linoleic acid was present in the second-highest concentration, at a minimum of 23.4% in the first harvest JAPC of Kalevala and a maximum of 26.9% in the second harvest JAPC of Alba. All of the analyzed JAPC samples exhibited a low concentration of unknown fatty acid, which comprised 0.3–0.6% of the total fatty acid content (Figure 1). Among the monounsaturated fatty acids (MUFAs), oleic acid (C18:1ω–9) was detected at a high value (6.6–11.6%), whereas the content of palmitoleic acid (C16:1ω–7) was significantly lower and ranged from 0.7% to 1.1% (Figure 1). The saturated fatty acids (SFA), myristic acid (C14:0), palmitic acid (C16:0), and stearic acid (C18:0) were also identified. Palmitic acid was the most abundant saturated component with no significant differences (16.4–17.9%) either between clones or time of harvest. The percent composition of myristic acid (2.5–6.9%) and stearic acid (1.5–1.8%) in the JAPC fractions were markedly lower than that of palmitic acid. Opposing tendencies were found for the oleic and myristic acid contents between the first and second harvests. The myristic acid content in JAPC was higher in the first harvest in all the three JA clones, while the oleic acid content was higher in the second harvest JAPC of Alba and Kalevala (Figure 1).

### 3.6. Screening JAPC Phytochemicals Using UHPLC-ESI-ORBITRAP-MS/MS

**Figure 3:**
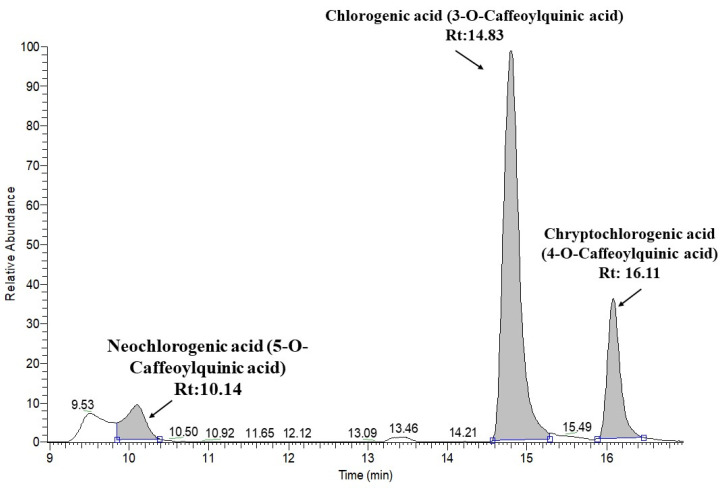
Extracted ion chromatogram of chlorogenic acid structural isomers.

The profiles of the phytochemicals in the JAPCs isolated from the JA clones Alba, Fuseau, and Kalevala, exhibited negligible differences between them. Up to 61 phytochemicals were defined, based on specific retention time, accurate mass, isotopic distribution, and fragmentation pattern, and by screening the following MS databases: Metlin, mzCloud, MoNA-MassBank of North America, and our own database. Table 3 indicates that phenolic compounds comprised a significant component of the compounds identified. Regardless of JA clones, three caffeoylquinic acid isomers: chlorogenic acid (3-*O*-caffeoylquinic acid), neochlorogenic acid (5-*O*-caffeoylquinic acid), and cryptochlorogenic acid (4-*O*-caffeoylquinic acid), respectively, were identified in the JAPCs with a characteristic [M−H]^−^ ion at *m*/*z* 353.0873. Considering the area of the peak of extracted ion chromatogram of isomers the 3-*O*-caffeoylquinic acid is the dominant one, while a lower ratio of neochlorogenic acid (5-*O*-caffeoylquinic acid) and cryptochlorogenic acid (4-*O*-caffeoylquinic acid) were detected (Figure 3). Additionally, three di-*O*-caffeoylquinic acid isomers ([M−H]^−^ ion at *m*/*z* 515.1190), four coumaroylquinic acid isomers ([M−H]^−^ ion at *m*/*z* 337.0924), and a 5-*O*-feruloylquinic acid ([M−H]^−^ ion at *m*/*z* 367.1029) were identified in the hydro-alcoholic extracted JAPC. The investigation also revealed a compound with a [M−H]^−^ ion at *m*/*z* 299.0767 in all of the JAPC extracts. The ion scan experiment of this ion showed corresponding fragment ions at *m*/*z* values of 137.0233; 113.0229; 93.0331; 85.0281; and 71.0122. After comparison with the databases, this compound was identified as salicylic acid 2-*O*-β-d-glucoside.

**Figure 4:**
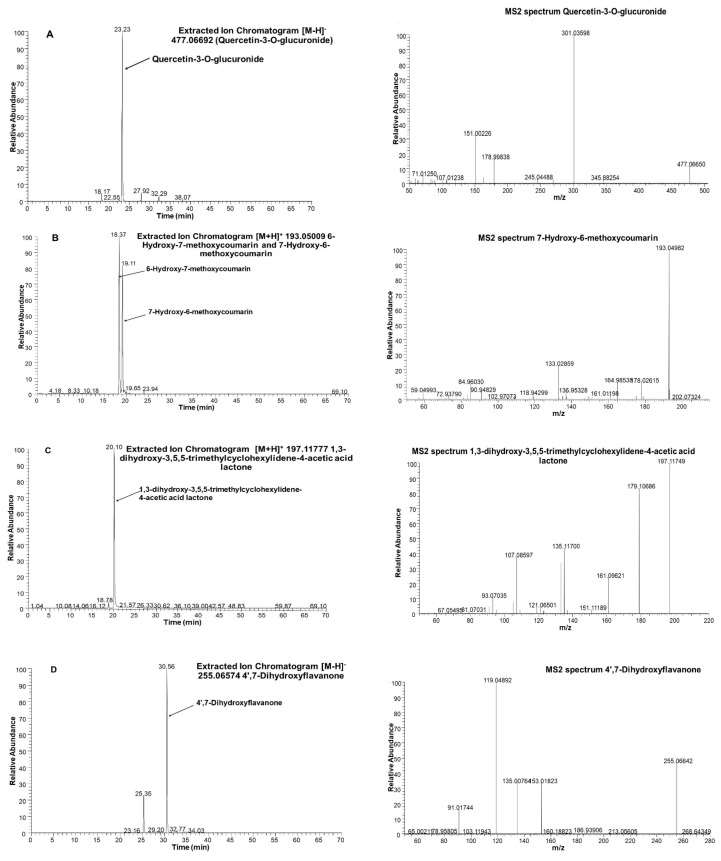
Extracted Ion Chromatograms (XIC) and MS spectra of selected phytoconstituents from Jerusalem artichoke leaf protein concentrate: (**A**): quercetin-3-*O*-glucuronide; (**B**): 7-Hydroxy-6-methoxycoumarin (Scopoletin); (**C**): 1,3-dihydroxy-3,5,5-trimethylcyclohexylidene-4-acetic acid lactone (Loliolide); and (**D**): 4′,7-Dihydroxyflavanone (Liquiritigenin).

Among flavonoids, isorhamnetin-3-*O*-glucoside with *m*/*z* 477.1033, kaempferol 3-glucuronide (kaempferol 3-*O*-β-d-glucopyranosiduronic acid) with *m*/*z* 461.0720, and astragaline (kaempferol 3-*O*-β-d-glucopyranoside) with *m*/*z* 447.0927 was found in the JAPC. However, to our knowledge, this is the first time glucuronide derivatives of isorhamnetin (isorhamnetin-3-*O*-glucuronide) and isoquercetin (quercetin 3-*O*-β-d-glucopyranoside) with *m*/*z* 463.0877 (Table 3 and Figure 4) have been identified. In addition to flavonols, most of the identified flavonoids belonged to the flavones. As far as we are aware, none of these has been identified previously in JAPC. For instance, we identified two dimethoxy-trihydroxyflavone isomers ([M − H]^−^ ion at *m*/*z* 329.0661), dimethoxy-tetrahydroxyflavone ([M − H]^−^ ion at *m*/*z* 345.0611), dihydroxy-methoxyflavone ([M − H]^−^ ion at *m*/*z* 283.0607), and trihydroxy-trimethoxyflavone ([M − H]^−^ at *m*/*z* 359.0767). Hymenoxin (5,7-dihydroxy-3′,4′,6,8-tetramethoxyflavone) at *m*/*z* 375.1080 and nevadensin (5,7-hydroxy-4′,6,8-trimethoxyflavone) at *m*/*z* 317.1389 were identified in positive ESI mode (Table 3). Within flavonoids, Butein (2′,3,4,4′-tetrahydroxychalcone) and kukulkanin B (3′-methoxy-2′,4,4′-methoxychalcone) which related to chalcones subgroup were identified. Finally, liquiritigenin (4′,7-dihydroxyflavanone; [M − H]^−^ at *m*/*z* 255.0657) was the only flavanone found in this study (Figure 4).

In addition to polyphenols, three different terpenes consistently appeared in the JAPC of the JA clones. Loliolide (1,3-dihydroxy-3,5,5-trimethylcyclohexylidene-4-acetic acid lactone) is a C_11_ monoterpenoid lactone, which was observed with a [M + H]^+^ ion at *m*/*z* 197.1178 (Figure 4). Dihydroactinidiolide as a volatile monoterpene with a [M + H]^+^ ion at *m*/*z* 181.1229, and 7-deoxyloganic acid isomer, an iridoid monoterpene with a [M − H]^−^ ion at *m*/*z* 359.1342, were recognized. Several proteinogenic amino acids were also identified (Table 3). In terms of vitamins, vitamin B molecules such as nicotinic acid (niacin; [M + H]^+^ ion at *m*/*z* 124.0399) and riboflavin ([M + H]^+^ ion at *m*/*z* 377.1461) were seen, while organic acids, i.e., malic acid and citric acid, and plant hormones such as indole acetic acid, were also identified in the JAPC.

## 4. Discussion

One important aspect of the biorefinery to become a competitive process is to produce at least one product of high value. The quantitative analysis of crude protein content of JAPC is a priority. The protein content of JAPC is influenced by plant type and also by the processing method. The average total protein content of the JAPC produced from Alba, Fuseau, and Kalevala was 33.4 m/m%; however, most of the isolated protein was found in the leaves, as these organs contain 3-fold higher total protein than the stem [23]. The JAPC comprised parenchyma tissues (80–87%) containing easily released cytoplasmic and chloroplast proteins such as Rubisco, which is of high nutritional value [24]. The time of harvest is critical to the quantity and quality of the JAPC produced from the aerial parts of the JA. Rashchenko [25] reported that the N content of older leaves is ~50% less than that in young leaves and Seiler [26] reported that the total protein content fell by 32.6% between the vegetative and flowering stages of JA growth. Knowing this, the shoots were harvested at the point of the maximum green leaf; ahead of senescence and before the bottom leaves turn dry. Ultimately, there was no significant difference in protein content between the two harvests.

In terms of an ideal protein source, the amino acid profile cannot be ignored, because among the 20 proteinogenic amino acids, nine cannot be synthesized by most animal species [20]. The content of these essential amino acids is, therefore, of particular interest. Among the green biomass fractions, the JAPC, as a dedicated protein enriched product for feed, was examined thoroughly. Several indispensable amino acids, i.e., lysine, isoleucine, leucine, methionine, and threonine, were present in high concentrations in the JAPC. However, even higher amino acid contents were found in JAPC by Rawate and Hill [27]; this may be attributed to different extraction methods and varieties. Additionally, the amino acid profiles exhibited minor differences between the two harvests, which may be due to differences in weather and plant age, as has previously been documented [11,25,26].

Considering the scientific literature about phytoconstituents of different JA organs, it was assumed that the green biomass-originated JAPC can be more than an alternative protein source. Qualitative analysis of phytochemicals in JAPC was performed by UHPLC-ESI-MS in both negative and positive ESI modes. The negative mode was used to identify flavonoid and phenolic acid (hydroxycinnamic acid and benzoic acid) derivatives, as it provided better sensitivity. The easy protonation of N in the positive mode made it suitable for identifying terpenes, amino acids, coumarins, and coumaroylquinic acids. 

Phenolic compounds are one of the largest groups of plant secondary metabolites. Among them, phenolic acids are an important subgroup and their presence is characteristic of the Asteraceae family. The most revealed phenolic acids are the mono- and di, and even tri-hydroxycinnamic acid (p-coumaric, caffeic, and ferulic acids) esters of quinic acids in the tuber and shoot organs of JA [15,17,19]. Our measurements confirmed 13 different “phenolic acids” from green biomass originated hydro-alcoholic extracted JAPC. The three structural isomers of caffeoylquinic acid were identified with a similar degree of ionization, and the same molecular weight and fragmentation pattern. Hence, the area of the peak of extracted ion chromatogram of isomers is comparable and the 3-*O*-caffeoylquinic acid seemed to be the dominant one (Figure 3). However, neochlorogenic acid (5-*O*-caffeoylquinic acid) displayed the lowest ratio. Chlorogenic acid (3-*O*-caffeoylquinic acid) is known as the most abundant isomer in plants, whereas cryptochlorogenic acid (4-*O*-caffeoylquinic acid) and neochlorogenic acid (5-*O*-caffeoylquinic acid) are present in much lower concentration [27]. Yuan et al. [15] cited 3-*O*-caffeoylquinic acid and 1,5-dicaffeoylquinic acid in high concentrations in JA leaves. However, Liang and Kitts [28] mentioned that 5-*O*-caffeoylquinic acid is the predominant isomer in fruits and vegetables. The presence of these phenolic acids is interesting from the aspect of both humans and animals, as several biological roles are attributed to caffeoylquinic acid isomers including antioxidant and antibacterial activities, hepato- and cardio-protection, anti-inflammatory and antipyretic activities, neuroprotection, anti-obesity, antiviral, and anti-hypertension activities, and central nervous system stimulation. Additionally, these compounds modulate lipid metabolism and glucose levels in both genetic metabolism-related disorders and healthy people [15,29]. Based on their health-promoting effects, caffeoylquinic acid isomers are increasingly recommended as natural and safe food additives, in place of synthetic antibiotics and immunity boosters.

**Figure 5:**
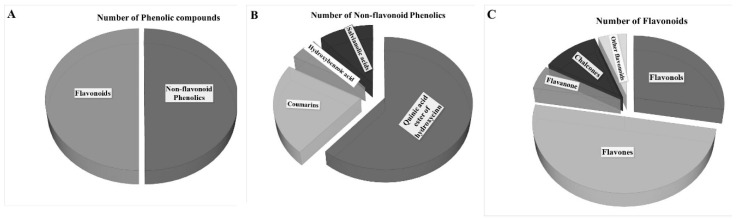
Identified phenolic compounds from Jerusalem artichoke leaf protein concentrate: (**A**) ratio of flavonoid and non-flavonoid phenolic compounds; (**B**) number of identified compounds within non-flavonoid phenolics subgroup; and (**C**) number of identified compounds within flavonoid phenolics subgroup.

The four different coumarins have also been revealed in the JAPC. Coumarins are widely distributed non-flavonoid polyphenols in the plant kingdom (Figure 5B). However, “simple coumarins” as coumarin subgroup is mainly present in the Asteraceae family. Therefore, each coumarin subclass-related compounds are used for the chemotaxonomic approach, too. Scopoletin and ayapin were already described in tubers of JA and assumed the presence of them in aerial part as in the case of *Helianthus annuus* [30]. Our measurement confirmed the presence of scopoletin along with isoscopoletin, 6-methyl coumarin, and fraxidin from green biomass originated product of JA. Some of simple coumarins are known as phytoalexins. At the same time, fraxidin and scopoletin have also shown potent antiadipogenic activity against the preadipocyte cell line in vitro assay systems [31]. Within non-flavonoid phenolics, two salvianolic acid derivatives and a salicylic acid-2-*O*-glucoside were also in detectable amounts.

Flavonoids are widespread secondary metabolites that occur as part of the phenolic constituents of plants. However, only a few of these have been described in the aerial part of JA including isorhamnetin glucoside, kaempferol glucuronide, and kaempferol-3-*O*-glucoside [14]. Based on the present qualitative analysis, 18 flavonoid compounds were revealed in the JAPC as green biomass originated product (Figure 5). Generally, cell vacuoles are the main storage places for soluble flavonoids. The JPAC is mostly made up of content released from the cytoplasm and vacuoles cell fractions, which may be the reason for the relatively high proportion of identified flavonoids. Within flavonoids, five flavonols were detected in the JAPC, in which all of them occurred as glycosides. Primarily, the solubility of flavonoids is due to their sugar substitutions. Among the sugars, glucose and glucuronic acid at a single position are probably the most common substituents [32]. The importance of flavonoid glucuronides is related to their health-promoting activities such as the anti-inflammatory and neuroprotective activities of quercetin-3-*O*-glucuronide [33]. Most of the identified flavonoid compounds belong to the flavones (Figure 5C). All of the flavone compounds were hydroxylated methoxyflavones, which contain one or more methoxy groups instead of a hydroxyl group on a flavone framework. The substitution of a methoxy group for a hydroxyl group in flavones has significant importance. One side the hydroxyl groups of flavones have free radical scavenging activity, but extensive conjugation of free hydroxyl groups to flavones results in low oral bioavailability; hence, they undergo rapid sulfation and glucuronidation in the small intestine and liver by phase II enzymes. Consequently, conjugated metabolites, but not the original compounds, can be found in plasma [34]. However, if one or more hydroxyl groups are capped by methylation, the substitution of a methoxy group by the hydroxyl group induces an increase in metabolic stability and improves transport and absorption. Considering the biological properties and chemical characteristics of hydroxyl and methoxy groups together, the hydroxylated methoxyflavones combine many advantages from both functional groups, improving their potential for application in human health [34]. Therefore, the presence of several hydroxylated methoxyflavones such as dimethoxy-trihydroxyflavone isomers, dimethoxy-tetrahydroxyflavone, dihydroxy-methoxyflavone, trihydroxy-trimethoxyflavone, hymenoxin, and nevadensin, increase the value of JAPC. 

From minor flavonoids, two chalcones were detected in JAPC. Butein (2′,3,4,4′-Tetrahydroxychalcone) is one of them which is widely biosynthesized in plants; however, no reference has been found citing it in JA. Based on preclinical studies, butein exhibits significant therapeutic potential against various diseases. In vitro and in vivo studies support that butein can suppress proliferation and trigger apoptosis in various human cancer cells with no or only minimal toxicity inducing in normal cells [35]. 

Liquiritigenin (4′,7-dihydroxyflavanone) as the only flavanone was measured in JA flowers by Johansson et al. [13]. Our results confirmed the presence of liquiritigenin in JAPC, too. Liquiritigenin is known to be a promising active estrogenic compound and is a highly selective estrogen receptor β agonist, which may be helpful to women who suffer from menopausal symptoms [36].

Three terpenes consistently appeared in the tested JAPC from all the JA clones. Loliolide, a C_11_ monoterpenoid lactone, is considered to be a photo-oxidative or thermally degraded product of carotenoids [37]. Similarly, we identified dihydroactinidiolide, a volatile monoterpenoid, which is a flavor component of several plants such as tobacco and tea. According to Yun et al. [38], thermal treatment induces the formation of dihydroactinidiolide from β-carotene. Kaszás et al. [8] confirmed that the green juice of the JAPC contains a marked number of carotenoids, which may be able partially to convert to loliolide or dihydroactinidiolide, causing the number of detectable terpenes to increase. Studies have confirmed that loliolide inhibits growth and germination, while also being phytotoxic, repelling leaf-cutter ants and having antitumor and antimicrobial activities in animals and microorganisms [37,39]. Dihydroactinidiolide has a carbonyl group that can react with nucleophilic structures in macromolecules, providing high potential reactivity to the molecules. It also shows cytotoxic effects against cancer cell lines [38]. The 7-Deoxyloganic acid isomer is the third terpene, which is known to be an intermediate in the secoiridoid pathway in plants.

The fatty acid and lipid contents of JA tubers have been reported by several authors [11,40]; however, little information is available about the fatty acid composition of its leaves and JAPC [41]. Recently, rapidly growing interest is for PUFAs, because humans and other mammals are incapable of synthesizing omega-6 and omega-3 PUFAs, due to the lack of Δ12 and Δ15 desaturase enzymes, which insert a cis double bond at the n-6 and n-3 positions [42]. Hence, linolenic and linoleic acids are essential nutrients converted from oleic acid in the endoplasmic reticulum of plant cells. Linolenic acid is the precursor of longer-chain PUFAs such as eicosapentaenoic acid (EPA: C20:5ω –3) and docosahexaenoic acid (DHA: C22:6ω –3), which can be synthesized in humans. Similarly, linoleic acid is an essential precursor to dihomo-γ-linoleic acid (DGLA: C20:3ω –6) and arachidonic acid (C20:4ω –6). As they are essential to life, linolenic and linoleic acids must be supplied to animals and humans through diet. In the JAPC of all JA clones, the highest contribution to the fatty acid profile was made by linolenic acid (38.6–42.7%) and linoleic acid (23.4–26.9%) as shown in Figure 1 and Figure 2. The correct proportions of linoleic and linolenic acids are emphasized by anthropological and epidemiological studies. The required ratio of omega-6 to omega-3 essential fatty acids is ~1:1, according to the evolutionary history of the human diet. In contrast, in the current Western diet, this ratio has shifted to 10–20:1, which is not beneficial to health and promotes the pathogenesis of many diseases [43]. We found a ratio of ~0.6:1 for omega-6 to omega-3 essential fatty acids in JAPC, which is very favorable and close to Paleolithic nutrition levels. 

Concerning the harvest time, Alba and Kalevalas’ JAPC exhibited higher oleic acid contents in the second harvest (when the nights were cooler). According to Barrero-Sicilia et al. [44], plants often respond to low temperature by increasing the levels of unsaturated fatty acids in the membrane and increasing membrane fluidity and stabilization. We found the opposing tendency in the saturated myristic acid (C14:0) contents of the JAPC, in which levels were higher in the first harvest (when the nights were warmer).

In summary, this study delivers deeper insights into JAPC originating from the fractionated green biomass of different JA clones focusing on its content of different phytochemicals that are potentially bioactive compounds and have several important uses. Future studies should investigate the anti-nutritional ingredients of JAPC, analyze the chemical composition of other fractions such as the brown juice and fiber, and calculate the economic viability of JA crops.

## 5. Conclusions

This paper discusses two important points related to JA. Firstly, we examined the potential production of leaf protein from its aerial parts. Secondly, we aimed to determine the quality of produced JAPC as a promising protein source that could be directed to human consumption and/or animal feeding. Biochemical analyses revealed that the JAPC is not only a good source of protein with a favorable amino acid composition but also a repository of essential fatty acids, flavonoid and non-flavonoid phytonutrients. The saturated palmitic acid (C16:0), stearic acid (C18:0), and the monosaturated form of stearic acid, oleic acid (C18:1ω–9), are often referred to as common fatty acids. They are biosynthesized in the plastids and partially incorporated into the cell and subcellular membranes [45]. The JAPC originates mainly from crushed cells of vegetative tissues containing membrane debris, which explains the relatively higher proportion of palmitic (16.4–17.9%) and oleic (6.6–11.6%) acids. Moreover, several important viable compounds were detected in JAPC. These compounds are known for their antibacterial, anti-inflammatory, and antipyretic activities, neuroprotection, anti-obesity, antiviral, and anti-hypertension activities, hepato- and cardio- protection, and central nervous system stimulation. However, the quantity and quality of the phytochemicals are specific to the species and can vary with the bioanalytical technology used. Hence, a quantitative analysis of identified phytocompounds needs to confirm the nutritional value of JAPC. Overall, the present results confirm that the green aerial parts of this underestimated plant can be a source of marketable products involving into green biorefinery concept. 

## Figures and Tables

**Table 1:** Aerial fresh biomass, dry mass, and total protein content of Jerusalem artichoke leaf protein concentrate (JAPC) isolated from green biomass of different clones.

Clones	Fresh Biomass Yield (kg m^−2^)		JAPC (g kg^−1^ Fresh Biomass)		Total Protein %	
	1st Harvest	2nd Harvest	1st Harvest	2nd Harvest	1st Harvest	2nd Harvest
Alba	5.0 ± 0.43 a	1.8 ± 0.22 b	31.9 ± 0.63 a	30.4 ± 0.59 a	35.3 ± 0.8 a	31.6 ± 0.8 b
Fuseau	5.2 ± 0.28 a	2.6 ± 0.19 ab	28.3 ± 0.04 a	28.8 ± 0.25 a	33.3 ± 0.9 a	35.2 ± 0.8 a
Kalevala	5.6 ± 0.65 a	2.8 ± 0.57 a	32.3 ± 0.53 a	28.0 ± 0.13 a	33.8 ± 0.7 a	33.4 ± 0.7 ab

Means followed by different letters in the same column show significant differences according to Duncan’s test at *p* < 0.05.

**Table 2:** Amino acid profile (m/m%) of Jerusalem artichoke leaf protein concentrate (JAPC) extracted from green biomass of different clones.

Amino Acid	1st Harvest			2nd Harvest		
	Alba	Fuseau	Kalevala	Alba	Fuseau	Kalevala
Lysine	2.32 ± 0.02 ^‡^ a	2.19 ± 0.02 c	2.25 ± 0.02 b	2.35 ± 0.03 c	2.54 ± 0.01 a	2.46 ± 0.02 b
Histidine	0.80 ± 0.20 a	0.71 ± 0.01 b	0.83 ± 0.03 a	0.72 ± 0.02 c	0.76 ± 0.02 b	0.82 ± 0.02 a
Isoleucine	1.72 ± 0.03 a	1.64 ± 0.02 b	1.77 ± 0.02 a	1.72 ± 0.02 bc	1.86 ± 0.02 a	1.78 ± 0.02 ab
Leucine	3.25 ± 0.05 b	3.08 ± 0.02 c	3.31 ± 0.01 a	3.19 ± 0.02 b	2.46 ± 0.02 c	3.30 ± 0.10 a
Phenylalanine	2.12 ± 0.02 b	1.96 ± 0.02 c	2.19 ± 0.01 a	2.03 ± 0.03 b	2.20 ± 0.10 a	2.18 ± 0.02 a
Methionine	0.87 ± 0.03 a	0.84 ± 0.02 a	0.79 ± 0.03 b	0.82 ± 0.02 b	0.95 ± 0.01 a	0.77 ± 0.02 c
Threonine	1.96 ± 0.01 b	1.87 ± 0.02 c	2.33 ± 0.03 a	1.95 ± 0.02 c	2.12 ± 0.02 b	2.33±0.03 a
Valine	2.05 ± 0.05 a	2.02 ± 0.02 a	2.06 ± 0.02 a	2.10 ± 0.02 b	2.34 ± 0.01 a	2.09 ± 0.01 b
Alanine	2.36 ± 0.05 a	2.20 ± 0.10 b	2.35 ± 0.02 a	2.32 ± 0.02 b	2.47 ± 0.02 a	2.34 ± 0.02 b
Arginine	2.08 ± 0.04 a	1.88 ± 0.02 b	1.86 ± 0.01 b	1.87 ± 0.02 c	1.97 ± 0.02 b	2.21 ± 0.01 a
Aspartic acid	3.81 ± 0.01 b	3.63 ± 0.03 c	4.23 ± 0.03 a	3.89 ± 0.02 b	4.23 ± 0.03 a	4.24 ± 0.04 a
Cysteine	0.24 ± 0.02 a	0.22 ± 0.02 a	0.22 ± 0.02 a	0.24 ± 0.02 ab	0.26 ± 0.02 a	0.23 ± 0.03 bc
Glycine	2.04±0.04 b	1.93 ± 0.03 c	2.13 ± 0.01 a	1.99 ± 0.01 b	2.14 ± 0.01 a	2.14 ± 0.02 a
Glutamic acid	4.29 ± 0.01 bc	4.14 ± 0.02 c	4.82 ± 0.02 a	4.38 ± 0.02 c	4.74 ± 0.02 b	4.79 ± 0.02 a
Proline	1.92 ± 0.03 b	1.82 ± 0.02 c	2.20 ± 0.10 a	2.04 ± 0.02 b	2.18 ± 0.01 a	2.19 ± 0.01 a
Serine	1.74 ± 0.04 b	1.67 ± 0.02 b	1.90 ± 0.10 a	1.77 ± 0.02 c	1.89 ± 0.01 b	1.93 ± 0.01 a
Tyrosine	1.48 ± 0.02 a	1.38 ± 0.02 c	1.46 ± 0.02 ab	1.42 ± 0.02 c	1.61 ± 0.01 a	1.55 ± 0.01 b
Ammonia	0.49 ± 0.01 ab	0.47 ± 0.02 b	0.52 ± 0.02 a	0.52 ± 0.02 a	0.48 ± 0.02 b	0.54 ± 0.02 a

^‡^ Standard deviation. Means followed by different letters in the same row and same harvest show significant differences according to Duncan’s test at *p* < 0.05.

**Table 3:** Chemical composition of Jerusalem artichoke leaf protein concentrate (JAPC) extracted from green biomass.

No.	Compound	Formula	Retention Time	Measured Mass (*m*/*z*)		Fragments 1	Fragments 2	Fragments 3	Fragments 4	Fragments 5
				[M + H]^+^	[M − H]^-^					
1	γ-Aminobutyric acid	C_4_H_9_NO_2_	1.25	104.07116		87.0446	86.0607	69.0342	58.0658	
2	Quinic acid	C_7_H_12_O_6_	1.27		191.05557	173.0447	171.0289	127.0388	93.0331	85.0280
3	Betaine (Trimethylglycine)	C_5_H_11_NO_2_	1.28	118.08681		59.0737	58.0659			
4	Malic acid	C_4_H_6_O_5_	1.33		133.01370	115.0024	89.0230	87.0075	72.9916	71.0123
5	Nicotinic acid (Niacin)	C_6_H_5_NO_2_	1.51	124.03986		96.0450	80.0501	78.0347		
6	Citric acid	C_6_H_8_O_7_	1.73		191.01918	173.0082	129.0182	111.0075	87.0073	85.0280
7	Neochlorogenic acid (5-*O*-Caffeoylquinic acid)	C_16_H_18_O_9_	10.14		353.08726	191.0557	179.0344	173.0448	135.0441	
8	Salicylic acid-2-*O*-glucoside	C_13_H_16_O_8_	13.56		299.07670	137.0234	113.0229	93.0331	85.0280	71.0123
9	Chlorogenic acid (3-*O*-Caffeoylquinic acid)	C_16_H_18_O_9_	14.83		353.08726	191.0556	179.0344	173.0443	161.0234	135.0441
10	Cryptochlorogenic acid (4-*O*-Caffeoylquinic acid)	C_16_H_18_O_9_	16.11		353.08726	191.0555	179.0344	173.0447	161.0232	135.0441
11	4-*O*-(4-Coumaroyl) quinic acid	C_16_H_18_O_8_	16.14		337.09235	191.0555	173.0447	163.0390	119.0489	93.0331
12	Vanillin (4-Hydroxy-3-methoxybenzaldehyde)	C_8_H_8_O_3_	16.22	153.05517		125.0600	111.0445	110.0366	93.0341	65.0393
13	5-*O*-(4-Coumaroyl)quinic acid	C_16_H_18_O_8_	17.38		337.09235	191.0556	173.0447	163.0391	119.0490	93.0332
14	Indole-3-acetic acid	C_10_H_9_NO_2_	17.98		174.05551	146.0601	144.0440	130.0651	128.0492	
15	4-*O*-(4-Coumaroyl)quinic acid cis isomer	C_16_H_18_O_8_	18.04		337.09235	191.0556	173.0447	163.0391	119.0489	93.0331
16	Isoscopoletin (6-Hydroxy-7-methoxycoumarin)	C_10_H_8_O_4_	18.33	193.05009		178.0264	165.0550	149.0598	137.0600	133.0287
17	5-*O*-Feruloylquinic acid	C_17_H_20_O_9_	18.42		367.10291	193.0503	191.0556	173.0447	134.0362	93.0331
18	Riboflavin	C_17_H_20_N_4_O_6_	19.03	377.14611		359.1352	243.0879	200.0824	172.0872	69.0342
19	Scopoletin (7-Hydroxy-6-methoxycoumarin)	C_10_H_8_O_4_	19.08	193.05009		178.0263	165.0546	149.0597	137.0601	133.0287
20	Azelaamic acid (9-Amino-9-oxononanoic acid)	C_9_H_17_NO_3_	19.21		186.11302	125.0959	97.0647			
21	6-Methylcoumarin	C_10_H_8_O_2_	19.44	161.06026		133.0651	115.0547	105.0704	91.0547	79.0549
22	5-*O*-(4-Coumaroyl)quinic acid cis isomer	C_16_H_18_O_8_	19.63		337.09235	191.0555	173.0446	163.0390	119.0491	93.0330
23	Indole-4-carbaldehyde	C_9_H_7_NO	19.67	146.06059		118.0655	117.0574	91.0548		
24	Fraxidin or Isofraxidin	C_11_H_10_O_5_	19.72		221.04500	206.0219	190.9983	163.0030		
25	Loliolide	C_11_H_16_O_3_	20.05	197.11777		179.1069	161.0962	135.1171	133.1015	107.0860
26	4-Hydroxy-3-methoxycinnamaldehyde (Coniferyl aldehyde)	C_10_H_10_O_3_	20.59	179.07082		161.0599	147.0442	133.0652	119.0495	55.0186
27	7-Deoxyloganic acid isomer	C_16_H_24_O_9_	22.36		359.13421	197.0815	153.0909	135.0805	109.0643	89.0230
28	Di-*O*-caffeoylquinic acid isomer 1	C_25_H_24_O_12_	22.61		515.11896	353.0884	191.0556	179.0342	173.0447	135.0441
29	Di-*O*-caffeoylquinic acid isomer 2	C_25_H_24_O_12_	22.77		515.11896	353.0884	191.0556	179.0342	173.0446	135.0440
30	Salvianolic acid derivative isomer 1	C_27_H_22_O_12_	22.80		537.10331	375.0705	201.0165	179.0343	161.0234	135.0440
31	Butein (2′,3,4,4′-Tetrahydroxychalcone)	C_15_H_12_O_5_	23.00	273.07630		255.0656	227.0699	209.0602	163.0391	137.0235
32	Quercetin-3-*O*-glucuronide	C_21_H_18_O_13_	23.26		477.06692	301.0359	178.9980	163.0028	151.0026	121.0281
33	Isoquercitrin (Hirsutrin, Quercetin-3-*O*-glucoside)	C_21_H_20_O_12_	23.47		463.08765	301.0358	300.0283	271.0253	255.0300	
34	Chrysoeriol-*O*-glucoside	C_22_H_22_O_11_	23.87		461.10839	299.0560	298.0484	270.0537	255.0292	227.0346
35	Salvianolic acid derivative isomer 2	C_27_H_22_O_12_	24.60		537.10331	375.0705	201.0166	179.0343	161.0236	135.0440
36	Di-*O*-caffeoylquinic acid isomer 3	C_25_H_24_O_12_	24.62		515.11896	353.0884	191.0557	179.0342	173.0447	135.0440
37	Azelaic acid	C_9_H_16_O_4_	25.05		187.09704	169.0863	143.1070	125.0959	123.0803	
38	Kaempferol-3-*O*-glucuronide	C_21_H_18_O_12_	25.18		461.07200	285.0410	229.0505	113.0231		
39	Apigenin-*O*-malonylglucoside	C_24_H_22_O_13_	25.21		517.09822	473.1116	269.0461	268.0376		
40	Astragalin (Kaempferol-3-*O*-glucoside)	C_21_H_20_O_11_	25.26		447.09274	285.0410	284.0331	255.0302	227.0350	
41	Isorhamnetin-3-*O*-glucoside	C_22_H_22_O_12_	25.48		477.10330	315.0524	314.0437	285.0406	271.0248	243.0292
42	Kukulkanin B (2′,4′,4-Trihydroxy-3′-methoxyxchalcone)	C_16_H_14_O_5_	25.50	287.09195		269.0810	241.0864	177.0548	145.0286	137.0235
43	Isorhamnetin-3-*O*-glucuronide	C_22_H_20_O_13_	25.70		491.08257	315.0517	300.0275	271.0249		
44	Dihydroactinidiolide	C_11_H_16_O_2_	27.16	181.12286		163.1119	145.1014	135.1171	121.1015	107.0860
45	Dimethoxy-tetrahydroxyflavone	C_17_H_14_O_8_	28.38		345.06105	330.0386	315.0153	287.0204	215.0347	178.9978
46	Dihydroxy-methoxyflavone	C_16_H_12_O_5_	29.89		283.06065	268.0381	267.0305	240.0427	239.0350	211.0396
47	Dimethoxy-trihydroxyflavone isomer 1	C_17_H_14_O_7_	30.09		329.06613	314.0439	299.0197	283.0869	271.0247	255.0913
48	Trihydroxy-trimethoxyflavone	C_18_H_16_O_8_	30.36		359.07670	344.0541	329.0307	314.0075	301.0358	286.0129
49	Dimethoxy-trihydroxyflavone isomer 2	C_17_H_14_O_7_	30.38		329.06613	314.0439	299.0201	283.0871	271.0252	253.0763
50	Liquiritigenin (4′,7-Dihydroxyflavanone)	C_15_H_12_O_4_	30.56		255.06574	153.0183	135.0077	119.0489	91.0175	
51	Hymenoxin (5,7,Dihydroxy-3′,4′,6,8-tetramethoxyflavone)	C_19_H_18_O_8_	32.11	375.10800		360.0840	345.0606	342.0736	330.0367	317.0659
52	Epiafzelechin trimethyl ether	C_18_H_20_O_5_	33.32	317.13890		167.0704	163.0755	155.0705	137.0598	121.0651
53	Nevadensin (5,7-Dihydroxy-4′,6,8-trimethoxyflavone)	C_18_H_16_O_7_	33.91	345.09743		330.0736	315.0501	312.0631	287.0554	
